# In light of cognitive flexibility and unconditional self-acceptance: the association between self-compassion and depression

**DOI:** 10.3389/fpsyg.2026.1750895

**Published:** 2026-10-26

**Authors:** Beyza Nur Turgut, Eyüp Çelik

**Affiliations:** Department of Guidance and Psychological Counseling, Sakarya University, Sakarya, Türkiye

**Keywords:** cognitive flexibility, depression, mediation analysis, self-compassion, unconditional self-acceptance

## Abstract

**Introduction:**

This study examined the relationship between self-compassion and depressive symptoms, focusing on the mediating roles of cognitive flexibility and unconditional self-acceptance. Although self-compassion is recognized as a protective factor against depression, the underlying psychological mechanisms remain insufficiently understood.

**Methods:**

A quantitative relational design was employed with a sample of 318 Turkish adults aged 18–65 years (76.1% female). Participants were recruited using convenience and snowball sampling methods, and data were collected via online surveys. Participants completed validated scales measuring self-compassion, depression, unconditional self-acceptance, and cognitive flexibility. Mediation analyses were conducted using the PROCESS macro (Model 4) in SPSS to test direct and indirect effects.

**Results:**

Correlation analysis indicated a significant negative relationship between self-compassion and depressive symptoms (*r* = −0.56, *p* < 0.001). Mediation analyses showed that cognitive flexibility significantly mediated this relationship, whereas unconditional self-acceptance demonstrated a weaker indirect effect.

**Discussion:**

Higher self-compassion was associated with lower depressive symptoms, partly through cognitive flexibility and, to a lesser extent, unconditional self-acceptance. These findings highlight the role of cognitive and self-evaluative processes in understanding the relationship between self-compassion and depression.

## Introduction

Depression has been recognized throughout human history and remains an important construct widely studied in the literature. Depression is a mood disorder characterized by persistent sadness, loss of interest or pleasure, cognitive impairments, and functional difficulties (Simon et al., 2024). Although these factors may vary across individuals, some factors may increase the risk of depression, whereas others may serve protective functions. When we look at the etiology, it is known that medical illnesses, loneliness, loss of a loved one, low socioeconomic level, unemployment, separation from spouse, loneliness, childhood traumas, difficulties in working life, problems in the family, and substance use cause depression (Ólafsdóttir et al., 2018; Levola et al., 2020; Kafes, 2021; Bischof et al., 2022; Fan et al., 2023). The prevalence of depression may increase as the number or intensity of contributing factors increases. According to the World Health Organization (2023), an estimated more than 300 million people worldwide are affected by depression, corresponding to approximately 4–5% of the global population. Its high prevalence worldwide and chronic course indicate that depression remains an important public health concern (Remes et al., 2021; Goodwin et al., 2022). Depression leads to significant functional impairments, making it difficult for individuals to cope with daily life. Depression is also associated with impaired functioning and interpersonal difficulties, and its prevalence varies according to gender and age (Fukuzaki and Takeda, 2022). This disorder, which can be seen at any age, occurs more frequently in middle age, especially between the ages of 25 and 50, and the lifetime prevalence rate is reported as 30% in women and 17% in men (Simon et al., 2024). Women are approximately twice as likely as men to experience depression. Didin and Yavuz (2022) stated that girls experience higher levels of depression than boys.

Previous studies have shown that depression is associated with various psychological and psychosocial variables, including emotion regulation difficulties, loneliness, life satisfaction, and self-esteem (Achterbergh et al., 2020; Kagan and Tleubayev, 2020; Cunningham et al., 2021; Dogan, 2021; Gao et al., 2022; Bhattacharya et al., 2023; Saldivia et al., 2023; Luo et al., 2024; Haehner et al., 2025). Examining depression in relation to psychological variables may help clarify factors associated with depressive symptoms.

Positive psychology has gained increasing attention in recent literature, focusing on individuals' strengths and wellbeing (Ergün, 2020; Kulbaş, 2021; Alam, 2022; Ciarrochi et al., 2022). Within this framework, self-compassion is considered an important construct associated with psychological growth and wellbeing (Pyszkowska, 2020). Rather than focusing solely on psychological difficulties, self-compassion involves a balanced and kind attitude toward one's own experiences, particularly in the face of personal shortcomings and distress. In this context, self-compassion may contribute to greater resilience and self-acceptance when individuals encounter psychological challenges. Self-compassion is conceptualized as an attitude that involves recognizing human imperfection and responding to oneself with understanding rather than self-criticism (Neff, 2023). According to Germer and Neff (2013), self-compassion consists of three interrelated components: self-kindness, common humanity, and mindfulness. These components reflect a balanced and non-judgmental awareness of personal experiences rather than over-identification with negative thoughts and emotions. Self-compassion promotes a balanced awareness of distressing experiences without suppression, denial, or over-identification (Neff, 2008; Germer and Neff, 2013).

Previous research has linked self-compassion to a range of positive psychological outcomes, including psychological wellbeing, emotional intelligence, resilience, and mindfulness, while demonstrating negative associations with depression and anxiety (Brophy et al., 2020; de Souza et al., 2020; Küçük, 2020; Pandey et al., 2021; Di Fabio and Saklofske, 2021; Tafarojei and Yousefi, 2021; Lou et al., 2022; Bailey et al., 2023; Li et al., 2024). These findings suggest that self-compassion may function as an important psychological resource associated with adaptive functioning and reduced psychological distress.

The understanding, compassion, and unconditional acceptance that individuals show toward themselves have a significant effect on their depression levels (Deniz and Sümer, 2010). It has been stated that individuals who have compassion and acceptance toward themselves, when they fail, do not criticize themselves harshly, but think that this situation is something that can happen to everyone, and they can approach it positively without making harsh judgments toward themselves (Neff, 2023). Self-compassion is protective in preventing depressive symptoms by providing individuals with a strong resilience against feelings of inadequacy and enabling them to be more attentive to themselves in difficult times (Warren et al., 2016). Previous studies consistently report a negative association between self-compassion and depressive symptoms (Altay, 2022; Çeştan, 2020; Han and Kim, 2023; Serrão et al., 2023; Wang et al., 2024). Similarly, unconditional self-acceptance prevents individuals from looking at themselves negatively, blaming themselves, and falling into depression (Acun Kapikiran and Kapikiran, 2010). In this context, Chamberlain and Haaga (2001b) stated that individuals who accept themselves unconditionally develop a healthier perspective and are more protected against depression and anxiety.

Gabrys et al. (2018) highlighted cognitive flexibility as an important variable in relation to depressive symptoms. Cognitive flexibility refers to the ability to adapt cognitive processes, modify thinking patterns, and generate alternative strategies in response to changing environmental demands (Braem and Egner, 2018; Dennis and Vander Wal, 2010). This capacity enables individuals to adjust to new situations and reinterpret experiences in a more adaptive way (Canas et al., 2006). Cognitive flexibility enables individuals to reinterpret distressing experiences and generate alternative responses to challenging situations, thereby supporting more adaptive cognitive and emotional processes (Dennis and Vander Wal, 2010; Marshall and Brockman, 2016). These processes are closely related to self-compassion and unconditional self-acceptance, as individuals with higher cognitive flexibility are more likely to adopt a non-judgmental and accepting attitude toward themselves and to recognize shared human experiences of suffering (Zhang et al., 2024).

On the other hand, lower cognitive flexibility may increase vulnerability to depression by limiting individuals' ability to reinterpret experiences and consider alternative perspectives (Dajani and Uddin, 2015; Yu et al., 2020). This restriction in cognitive processing can lead individuals to focus rigidly on specific interpretations, which is associated with increased depressive symptoms. Conversely, higher cognitive flexibility facilitates adaptive thinking, supports effective coping with stressful situations and interpersonal difficulties, and is linked to lower levels of depression (Fukuzaki and Takeda, 2022; Güler, 2022). Overall, cognitive flexibility appears to play a protective role in emotional adjustment and depressive symptomatology.

Otherwise, when individuals fail to recognize their strengths, their ability to show compassion to themselves and their level of self-acceptance decrease, and their tendency toward depression increases (Popov, 2019). Furthermore, unconditional self-acceptance enhances self-compassion and strengthens its protective effect against depressive symptoms (Crisan et al., 2023). Indeed, unconditional self-acceptance has been shown to support self-compassion in protecting against depression (Chamberlain and Haaga, 2001a). Therefore, unconditional self-acceptance may represent an important mechanism linking self-compassion to lower depressive symptoms.

Individuals with high cognitive flexibility have been found to have higher problem-solving abilities, are more likely to forgive themselves for mistakes they make, and are more compassionate toward themselves (Martin et al., 2011). Neff et al. (2007) stated that cognitive flexibility helps individuals evaluate themselves positively and adopt an accepting attitude toward themselves. However, individuals lacking cognitive flexibility are at risk for depression because they are unable to accept themselves with their shortcomings and mistakes, are unable to approach themselves compassionately, and are unable to adapt to new processes (Çeştan, 2020; Reis, 2020). In other words, it is thought that high cognitive flexibility facilitates flexible thinking in the face of negative life events, supports individuals in showing compassion to themselves, and thus prevents the risk of developing depressive symptoms (Marshall and Brockman, 2016). Therefore, cognitive flexibility may function as a mediating mechanism through which self-compassion is associated with lower depressive symptoms.

Consistent with positive psychology and self-compassion theory, self-compassion can be conceptualized as an adaptive psychological resource that may reduce depressive symptoms by promoting more flexible cognitive processing and greater unconditional self-acceptance. Self-compassion can be associated with lower depressive symptoms by supporting emotion regulation processes, cognitive and self-evaluation mechanisms. Individuals with high levels of self-compassion tend to be more emotionally accommodating when experiencing personal failure and distress, approaching themselves with less self-criticism and acting with mindful awareness (Viskovich and De George-Walker, 2019). This approach may support cognitive flexibility by enabling individuals to re-evaluate negative experiences and adopt more adaptive perspectives in stressful situations. Similarly, self-compassion can promote unconditional self-acceptance by reducing the tendency to base self-worth on performance, failure, or external approval. In turn, both cognitive flexibility and unconditional self-acceptance have been associated with lower depressive symptoms through less rumination, self-criticism, and psychological rigidity (Popov, 2019; Faustino et al., 2020; Jouybari and Ghoreishi, 2025). Accordingly, in this study, cognitive flexibility and unconditional self-acceptance are conceptualized as potential pathways that can explain the relationship between self-compassion and depression.

The current study aimed to examine the mediating roles of unconditional self-acceptance and cognitive flexibility in the relationship between self-compassion and depression. Although previous research has linked these variables to depressive symptoms, limited evidence has examined them simultaneously within a single model. Investigating these relationships among Turkish adults may provide culturally relevant insight into how self-related cognitive and emotional processes are associated with depressive symptoms. In collectivist cultural contexts, self-acceptance and emotional coping processes may be shaped by interpersonal expectations and social evaluation.

The hypotheses of the research are as follows:

1. H_1_: Self-compassion is significantly and negatively associated with depression.
2. H_2_: Unconditional self-acceptance may statistically account for the association between self-compassion and depression.
3. H_3_: Cognitive flexibility may statistically account for the association between self-compassion and depression.

To test the proposed hypotheses, the following methodology was employed.

## Methods

In the current study, a quantitative research design, the relational screening model, was used to explore the relationships among depression, self-compassion, unconditional self-acceptance, and cognitive flexibility. Relational screening models are studies conducted to reveal the relationship between two or more variables and to determine the degree and direction of the relationship (Karasar, 2014). The study examined the mediating roles of unconditional self-acceptance and cognitive flexibility in the relationship between depression and self-compassion. A multiple mediation model was used to test direct and indirect effects.

### Participants

The study group consisted of 318 individuals between the ages of 18 and 65 (*M* = 31.16, *SD* = 9.89). The sample was selected using a convenience sampling method that met the inclusion criteria for participants who were willing to participate in the study. Participation in the study was voluntary. Three individuals who participated in the study but were under the age of 18 were excluded from the study group, resulting in a dataset of 318, down from 321. Snowball sampling was used to collect data from 318 Turkish adults. Convenience and snowball sampling methods were used to facilitate participant recruitment and increase participant reach through online networks, allowing access to a broader adult sample during the data collection process. The sample consisted of 242 females (76.1%) and 76 males (23.9%). 162 of the participants were single (50.9%) and 156 (49.1%) were married. Among the participants, 3 people (0.94%) were primary school graduates, 7 people (2.2%) were secondary school graduates, 37 people (11.6%) were high school graduates, 227 people (71.4%) were undergraduate graduates, and 44 people (13.8%) had postgraduate (master's-doctoral) education.

Ethical approval for this study was obtained from the Education Research and Publication Ethics Committee of [Sakarya University]. The study was approved at the committee meeting held on April 9, 2025 (Meeting No. 44, Decision No. 58).

### Measures

The Depression Anxiety Stress Scale-21 (DASS-21) is a 21-item self-report instrument developed by (Lovibond and Lovibond 1995). The Turkish adaptation of the scale was conducted by Yilmaz et al. (2017). The scale is a four-point Likert-type instrument ranging from 0 (“did not apply to me at all”) to 3 (“applied to me very much or most of the time”). It consists of three subscales: depression, anxiety, and stress, each containing seven items. In this study, raw (unmultiplied) scores were used; therefore, subscale scores range from 0 to 21. Cronbach's alpha coefficients were 0.86 for depression, 0.79 for anxiety, and 0.80 for stress. Confirmatory factor analysis supported the original three-factor structure (RMSEA = 0.06, CFI = 0.93, GFI = 0.92). Evidence for criterion validity was supported by significant correlations among the subscales (Yilmaz et al., 2017).

**Self-Compassion Scale:** The self-compassion scale developed by Neff (2003) was adapted into Turkish by Deniz et al. (2008). The original version consists of 26 items and 6 sub-dimensions. The self-compassion scale, adapted to Turkish by removing two items from the original, consists of a total of 24 items and exhibits a single-factor structure. The scale is a five-point Likert-type scale ranging from 1 “almost never” to 5 “almost always.” The internal consistency coefficient of the scale was 0.89, and the test-retest correlation was 0.83. The fit indices obtained from confirmatory factor analysis (CFA) indicate that the model overlaps the data at an acceptable level. Furthermore, criterion validity was supported by examining the scale's correlations with similar scales (Deniz et al., 2008).

**Unconditional Self-Acceptance Scale:** The unconditional self-acceptance scale developed by Chamberlain and Haaga (2001a) was adapted into Turkish by Acun Kapikiran and Kapikiran (2010). The Turkish adaptation of the original 20-item scale consists of 19 items. The scale, which consists of two sub-dimensions: conditional self-acceptance and unconditional self-acceptance, is a seven-point Likert-type scale ranging from 1 (almost always not true) to 7 (almost always true). The Cronbach's alpha value of the scale, which explains 36.77% of the total variance, is 0.76, the test-retest correlation is *r* = 0.62, and the item-total score correlations range from 0.40 to 72. Furthermore, the scale's significant and expected correlations with other scales measuring similar concepts support criterion validity (Acun Kapikiran and Kapikiran, 2010).

**Cognitive Flexibility Scale:** The cognitive flexibility scale developed by Martin and Rubin (1995) was adapted into Turkish by Altunkol (2011). The 12-item scale is a six-point Likert-type scale ranging from 1 (strongly disagree) to 6 (strongly agree). The validity of the scale was tested using both construct validity and criterion-related validity methods. Factor analysis conducted to assess construct validity revealed a unidimensional structure for the scale. Criterion-related validity revealed significant correlations with psychological wellbeing, problem solving, and adaptation levels, which are thought to be related to cognitive flexibility. The Cronbach alpha value of the scale was found to be 0.81, and it was seen that it met the validity criteria as a result of criterion-related validity and construct validity studies (Altunkol, 2011).

Permissions for the scales described above were obtained by the researcher.

### Procedure

In the current study, data were collected through an online survey design. The questionnaire was created using Google Forms to ensure easy accessibility and efficient data collection. Prior to data collection, the survey link was reviewed and finalized by the researchers. The survey was initially distributed by the researchers through multiple online channels, including email and social media platforms such as WhatsApp, Facebook, and Instagram. These platforms were selected to reach a diverse adult population in a time- and cost-efficient manner. In addition to initial distribution, a snowball sampling technique was employed to increase participant reach. Participants who completed the survey were asked to share the survey link with other individuals who met the inclusion criteria (i.e., being 18 years of age or older and voluntarily agreeing to participate). This approach allowed the study to access a wider pool of respondents beyond the initial networks of the researchers. Participation in the study was entirely voluntary and anonymous. At the beginning of the survey, participants were presented with an informed consent form explaining the purpose of the study, confidentiality of responses, and their right to withdraw at any time without penalty. Only participants who provided informed consent were able to proceed to the questionnaire. Ethical approval for the study was obtained from the relevant institutional ethics committee prior to data collection. No personally identifiable information was collected, and all responses were stored securely and used solely for research purposes.

### Data analysis

In the current study, Pearson correlation analysis was conducted to examine the relationships among depression, self-compassion, unconditional self-acceptance, and cognitive flexibility. Prior to regression analyses, assumptions of normality and linearity were evaluated, and skewness and kurtosis values were examined. The results indicated that the data met the assumptions for regression analysis. The mediating roles of unconditional self-acceptance and cognitive flexibility in the relationship between self-compassion (X) and depression (Y) were tested using SPSS PROCESS macro (Model 4; Hayes, 2013). CF and unconditional self-acceptance were examined as parallel mediators in a multiple mediation model to estimate direct and indirect effects. Moreover, a *post hoc* sensitivity power analysis was conducted using G^*^Power 3.1 to assess sample size adequacy. With *N* = 318, α = 0.05, and power = 0.80, the study was sufficiently powered to detect small effect sizes in the proposed mediation model. The minimum detectable effect size for a multiple regression model with three predictors was approximately *f*^2^ = 0.03–0.04, indicating adequate statistical sensitivity according to Cohen's criteria (Table 1).

**Table 1:** Descriptive statistics of the variables.

Variables	*N*	Min.	Max.	Means	*SD*	Skew.	Kurt.	VIF	Tolerance
Depression	318	0.00	21.00	6.47	5.77	0.67	−0.55		
Self-compassion	318	27.00	119.00	78.88	18.56	−0.09	−0.43	1.789	0.559
Self-acceptance	318	28.00	123.00	78.92	14.58	−0.00	0.24	1.781	0.561
Cognitive flexibility	318	29.00	64.00	50.55	6.37	−0.18	−0.24	1.176	0.850

DASS-21 subscale scores are presented as raw (unmultiplied) scores ranging from 0 to 21.

## Results

Relationships between variables were calculated using the Pearson Correlation Coefficient and the results are shown in Table 2.

**Table 2:** The relationships between variables.

Variables	1	2	3	4
1 = Depression	1			
2 = Self compassion	−0.56^**^	1		
3 = Self-acceptance	−0.45^**^	0.65^**^	1	
4 = Cognitive flexibility	−0.41^**^	0.41^**^	0.37^**^	1
M				
SD				

^**^p < 0.01.

### Mediation analysis

As shown in Table 3, simple linear regression prior to the mediation analysis indicated that self-compassion significantly predicted depression (β = −0.18, 95% CI: −0.20 to −0.14; *p* < 0.001). The mediating roles of unconditional self-acceptance and cognitive flexibility in the relationship between self-compassion and depression were then examined.

**Table 3:** Regression coefficients, standard errors, and significance tests for the regression model.

Predictor	Coeff.	*SE*	*p*	*F*	*R* ^2^
Constant	20.28	1.17	0.0000	146.30	0.32
Self-compassion	−0.18	0.01	< 0.001		

The mediating roles of unconditional self-acceptance and cognitive flexibility in the relationship between self-compassion and depression were tested with the mediation model, and the results are presented in Table 4.

**Table 4:** The serial multiple mediational model coefficients.

Predictors		Consequent									
		Self-acceptance			Cognitive Flexibility				Depression		
	Path	Coeff.	*SE*	*p*	Coeff.	*SE*	*p*		Coeff.	*SE*	*p*
Self-compassion	*a*	0.51	0.03	< 0.001	0.10	0.02	< 0.001	*c*′	−0.13	*0.02*	< 0.001
Self-acceptance		–	–	–	0.08	0.03	< 0.001	*b*_1_	−0.05	0.02	0.05
Cognitive flexibility		–	–	–	–	–	–	*b*_2_	−0.18	0.05	< 0.001
Constant	*i*_1_				36.24	1.80	< 0.001	*i*_2_	29.44	2.19	< 0.001
		*R*^2^ = 0.42			*R*^2^ = 0.19				*R*^2^ = 0.36		
		*F*_(1, 316)_ = 232.42, *p=* < 0.001			*F*_(2, 315)_ = 37.19, *p=* < 0.001				*F*_(3, 314)_ = 60.11, *p* = < 0.001		

Italicized values indicate statistically significant regression coefficients (*p* < 0.05).

The mediation model is presented in Figure 1, where self-compassion is the independent variable, depression is the dependent variable, and unconditional self-acceptance and cognitive flexibility are parallel mediators.

**Figure 1:**
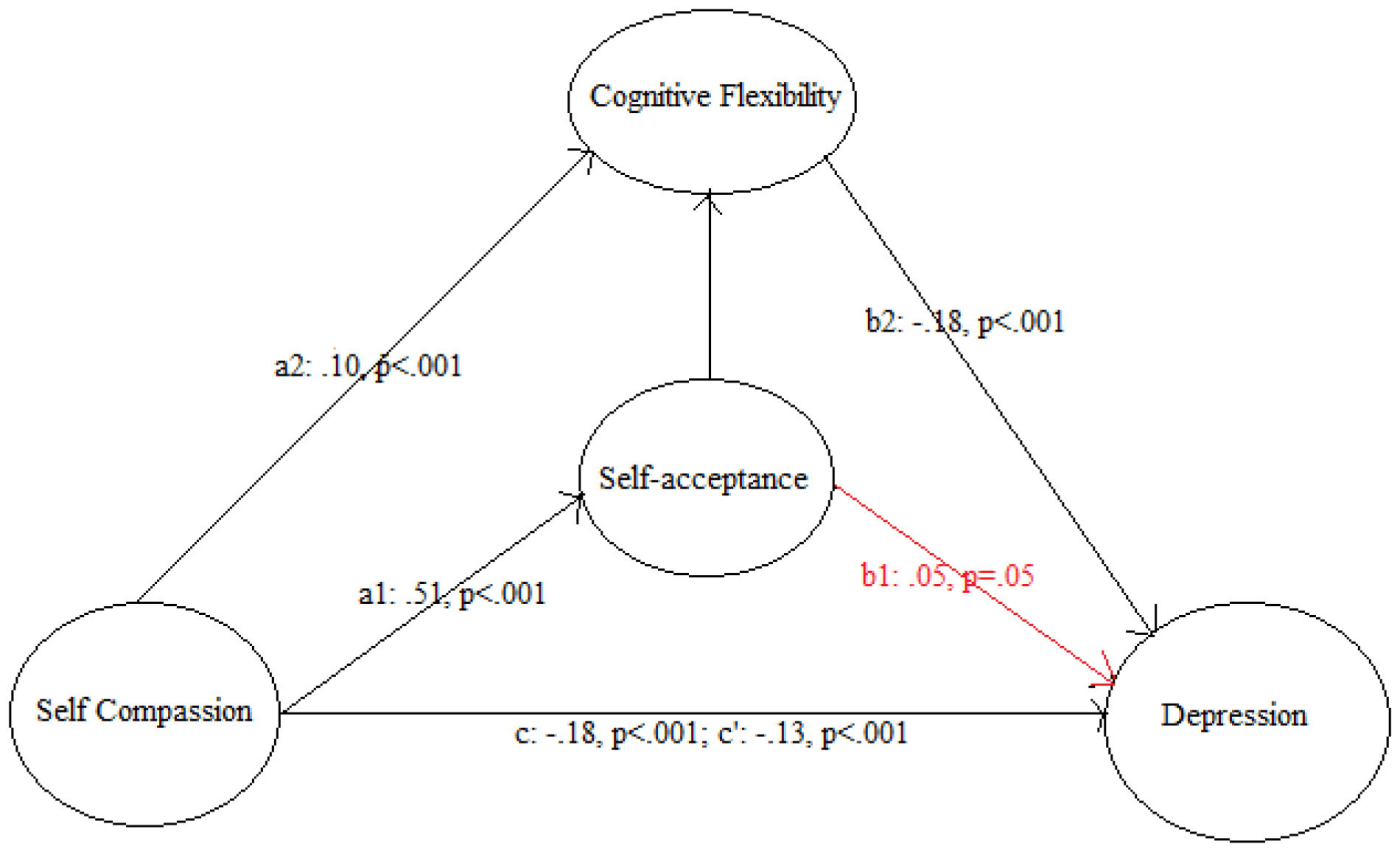
The mediating role of unconditional self-acceptance and cognitive flexibility.

As shown in Figure 1 and Table 4, the result of the mediation analysis showed that cognitive flexibility (Coeff. = 0.18, 95% CI: −0.28 to −0.10; *p* < 0.001) significantly predicted depression, but self-acceptance (Coeff. = −0.05, 95% CI: −0.09 −0.00; *p* = 0.05) didn't significantly predict depression. Self-compassion predicted the depression (Coeff. = 0.13, 95% CI: −0.16 to −0.09; *p* < 0.001), cognitive flexibility (Coeff. = 0.10, 95% CI: 0.06 −0.15; *p* < 0.001), and self-acceptance (Coeff. = 0.51, 95% CI: 0.45 −0.58; *p* < 0.001) significantly. Self-acceptance significantly predicted the cognitive flexibility (Coeff. = 0.08, 95% CI: 0.02 −0.14; *p* < 0.01). Furthermore, cognitive flexibility decreased the effect of self-compassion on depression (from −0.18 to −0.13). Finally, the relationship between self-compassion and depression was mediated by cognitive flexibility.

In the mediation analysis, the indirect, total, and direct effects of self-compassion on depression, mediated by unconditional self-acceptance and cognitive flexibility, were examined and presented in Table 5.

**Table 5:** Direct and indirect effects of self-compassion on depression via unconditional self-acceptance and cognitive flexibility.

Path	Effect	*SE*	LLCI	ULCI
Direct influence	−0.13	0.02	−0.16	−0.09
Indirect effect	−0.05	0.01	−0.08	−0.02
Ind1: Self-compassion → self-acceptance → depression	−0.02	0.01	−0.05	0.00
Ind2: Self-compassion → cognitive flexibility → depression	−0.02	0.01	−0.03	−0.01
Ind3: Self-compassion → self-acceptance → cognitive flexibility → depression	−0.01	0.00	−0.01	−0.00
Total effect	−0.18	0.01	−0.20	−0.15

Ind, indirect effect.

As seen in Table 5, the indirect effect of self-compassion on depression through cognitive flexibility was statistically significant, whereas unconditional self-acceptance demonstrated a weaker mediating effect. Furthermore, self-compassion continued to have a significant direct effect on depression after the mediators were included in the model, indicating partial mediation.

## Discussion

The current study examined the mediating roles of unconditional self-acceptance and cognitive flexibility in the relationship between depression and self-compassion. The findings indicate a significant negative relationship between depression and self-compassion. This finding suggests that individuals with high self-compassion are at less risk for depression. This result supports previous research results (Çeştan, 2020; Adie et al., 2021; Hughes et al., 2021; Altay, 2022; Egan et al., 2022; Etemadi Shamsababdi and Dehshiri, 2024). Self-compassion is defined as individuals having a kind, understanding, and non-judgmental attitude toward themselves (Neff, 2023). Having high self-compassion prevents individuals from developing a critical attitude toward themselves as a result of negative events experienced, enables them to cope with their negative emotions, thus reducing the risk of developing depressive symptoms (Abdollahi et al., 2020; Han and Kim, 2023). Germer and Neff (2020) stated that individuals with self-compassion are less affected by troublesome processes, are more resilient to psychological disorders, and have higher levels of happiness and life satisfaction. Especially when mistakes and interpersonal problems occur in life, a more understanding attitude by preventing individuals from being cruel to themselves allows them to experience their emotions in a balanced way and protect themselves against mental disorders (Brophy et al., 2020). However, the three components of self-compassion—self-understanding, common humanity, and awareness—are also effective in preventing the development of depression (Germer and Neff, 2020). When the literature is examined, it has been revealed that individuals with high self-compassion levels have reduced anxiety and stress levels along with depression, and self-compassion contributes to an increase in the psychological wellbeing of individuals (Ko et al., 2018; de Souza et al., 2020; Kroshus et al., 2021). High self-compassion allows individuals to accept themselves, to recognize their own feelings and thoughts objectively, to improve their self-esteem, and to develop healthy coping skills for their problems (Tran et al., 2022). In this way, self-compassion may be considered a psychological resource that is associated with lower depressive symptoms and may inform intervention approaches targeting emotional wellbeing. However, some researchers have suggested that the strength of the association between self-compassion and depressive symptoms may vary across populations and cultural contexts, indicating that the protective role of self-compassion may depend on contextual and individual factors (MacBeth and Gumley, 2012; Neff and Germer, 2013).

Other findings of the study revealed a significant negative correlation between depression and unconditional self-acceptance and cognitive flexibility. The current results support research in the literature (Guo et al., 2022; Naderi et al., 2023; Altan-Atalay and Boluvat, 2024). In light of this finding, increased depressive symptoms may be associated with lower levels of self-acceptance and cognitive flexibility. When individuals accept themselves unconditionally, without depending on any external process, they develop a more resilient personality toward distressing processes and are less likely to experience depressive symptoms (Chamberlain and Haaga, 2001a). Indeed, Hilbert et al. (2019) stated that the most important way to protect mental health is for individuals to accept themselves free from problematic life events and mistakes and to develop self-esteem. Individuals who value themselves conditionally feel worthless when faced with life's challenges, which can lead to symptoms of depression (Sanghvi et al., 2023). Furthermore, individuals who are cognitively inflexible are also more likely to experience depression (García-Fernández et al., 2025). Cognitive flexibility is defined as the ability to develop alternative solutions to problems, to think flexibly, and to develop a constructive and realistic mindset (Dajani and Uddin, 2015). In this context, individuals with cognitive flexibility skills can think more constructively about problems, allowing their depression levels to decrease (Fukuzaki and Takeda, 2022). Similarly, Gao et al. (2025) stated that a high level of cognitive flexibility is a general factor that reduces the likelihood of experiencing depression and facilitates adaptation to changing processes in daily life. The ability of cognitive flexibility to support adaptation, promote healthier interpretations of experiences, and facilitate flexible responses, together with the role of unconditional self-acceptance in enhancing self-evaluation, may represent protective processes against depressive symptoms. However, these protective processes may not operate in the same way across all individuals and contexts. Some studies have suggested that the associations between cognitive flexibility, self-acceptance, and depressive symptoms may vary depending on individual and contextual factors, such as psychological characteristics and environmental conditions (Dajani and Uddin, 2015; Gabrys et al., 2018; Chamberlain and Haaga, 2001a,b).

Other findings of the current study suggest that the relationship between self-compassion and depressive symptoms may be partially explained by cognitive flexibility and, to a lesser extent, unconditional self-acceptance. It is stated that one of the factors supporting self-compassion is unconditional self-acceptance (Zou et al., 2025). Unconditional self-acceptance is the acceptance of individuals without the need for success, approval from others, or appreciation (Yilmaz Bingöl and Vural Batik, 2018). Unconditional self-acceptance may contribute to the development of self-compassion by increasing individuals' awareness that they are valuable because of their existence (Faustino et al., 2020; Crisan et al., 2023). However, unconditional self-acceptance, like self-compassion, is a fundamental element of being a psychologically healthy individual (Muris and Otgaar, 2023). Because people with high levels of self-compassion are more understanding of themselves, they have no difficulty accepting their flaws (Faustino et al., 2020). On the other hand, individuals with unconditional self-acceptance accept themselves without judgment, allowing them to understand their personalities and thus feel better mentally (Zipagan and Galvez Tan, 2023). Kicali (2015) noted that unconditional self-acceptance and self-compassion, which have a self-esteem-enhancing function, are linked to lower negative affect. Individuals with higher levels of unconditional self-acceptance may demonstrate greater resilience in coping with difficulties and adopt a kinder attitude toward themselves, which may be associated with lower depressive symptoms. As a matter of fact, there are studies in the literature that support the findings of the current research showing that self-respect, acceptance and understanding are effective in preventing mental disorders such as depression (Moksnes et al., 2016; Dinger et al., 2017; Bae et al., 2021; Sayin and Karaaziz, 2024; Yilmaz, 2024). Therefore, the relationship between self-compassion and lower depressive symptoms may be partly explained by unconditional self-acceptance. Interventions aimed at enhancing self-compassion and unconditional self-acceptance may contribute to reducing vulnerability to depressive symptoms.

Cognitive flexibility appears to play a more prominent mediating role in the relationship between self-compassion and depressive symptoms. Cognitive flexibility is the ability to accept changing circumstances, evaluate options, and increase problem-solving skills (Dennis and Vander Wal, 2010). Cognitive flexibility, which facilitates adaptation to new processes, reveals alternative ways of thinking, enables individuals to forgive themselves when they make mistakes, and paves the way for them to develop self-compassion (Martin et al., 2011). However, cognitive flexibility has been identified as positively associated with psychological health (Yelpaze, 2020). When the literature is examined, it has been revealed that cognitive flexibility is effective in increasing wellbeing and reducing depression (Yazar and Meterelliyoz, 2019; Yu et al., 2020; Asici and Sari, 2021; Circir and Tagay, 2024; García-Fernández et al., 2025). It can be stated that cognitive flexibility is of may play an important role in coping with distressing events and plays an active role in preventing mental disorders. While individuals who are cognitively flexible and can consider different options adapt more quickly to the challenges in life, individuals who cannot think flexibly are more likely to develop pathological reactions and experience psychological problems (Okan Er and Deniz, 2022). When viewed within the framework of Beck's cognitive model, cognitive flexibility is an important factor in the process of revealing and evaluating the real and unconstructive cognitions of individuals with depressive symptoms (Aslan and Türk, 2022). It has been shown that individuals with flexible thinking can more easily transform their negative automatic thoughts into constructive thoughts, are more understanding toward themselves, and thus, their depression symptoms decrease (Curtiss and Klemanski, 2014; Yadavaia et al., 2014). Indeed, Topkaya et al. (2022) stated that the effect of self-compassion in reducing depression may be mediated by cognitive flexibility. Similarly, it has been determined that self-compassion and cognitive flexibility are interrelated and that they are effective together in reducing the level of negative self-esteem and depression (Güler, 2022). Therefore, improving cognitive flexibility may be beneficial in psychological interventions aimed at reducing depressive symptoms by supporting self-compassion processes. At the same time, the mediating role of cognitive flexibility should be interpreted cautiously, as previous findings have suggested that its contribution may depend on other cognitive and emotional processes involved in depression (Dajani and Uddin, 2015; Gabrys et al., 2018; Yu et al., 2020). Accordingly, the present findings suggest that cognitive flexibility and unconditional self-acceptance may represent potential pathways through which self-compassion is associated with depressive symptoms. Both cognitive flexibility and unconditional self-acceptance may function as cognitive and self-evaluative mechanisms underlying this association.

### Limitations

The study has some limitations. Firstly, it is a cross-sectional study. Because the study was conducted in a cross-sectional manner, the variables cannot be interpreted within the framework of a causal design. In this context, the narrower scope of the topic affected the selected sample and the generalizability of the results. Data for the study were collected via an online survey. In this context, the representativeness of the sample may be limited since the study does not include people who cannot access online platforms. Research data were collected based solely on participants' responses to survey questions. This situation may cause participants to present themselves in a socially acceptable and positive manner rather than providing realistic information about themselves, thus creating limitations in terms of the accuracy of the data obtained. Furthermore, the sample was predominantly female (76.1%) and highly educated (85.2% holding at least a bachelor's degree), which may limit the generalizability of the findings. Given evidence of gender differences in depression prevalence, this imbalance should be considered when interpreting the results. In addition, the broad age range of the sample (18–65) may have introduced age-related differences in depressive experiences and psychological processes.

### Implications for future research

The current study used a cross-sectional design. As a result of this cross-sectional design, causal relationships between variables could not be revealed. For this reason, this research can be conducted with longitudinal and experimental designs. In this study, unconditional self-acceptance and cognitive flexibility were used as mediating variables. Future research could examine different concepts as mediating variables. In addition, since the study sample is limited to a certain age group and culture, conducting the study in different age groups and cultural groups may increase its generalizability. Future studies may consider controlling for potential covariates such as age, gender, and education level. In terms of practice, it may be beneficial to develop psychoeducation programs aimed at increasing individuals' self-compassion, unconditional self-acceptance, and cognitive flexibility levels and to offer them as a preventive or supportive intervention for individuals at risk of depression. Future research may also examine the subdimensions of self-compassion to provide a more detailed understanding of their differential relationships with depression and related psychological constructs.

## Data Availability

The raw data supporting the conclusions of this article will be made available by the authors, without undue reservation.
